# Genetic and Epigenetic Biomarkers Linking Alzheimer’s Disease and Age-Related Macular Degeneration

**DOI:** 10.3390/ijms25137271

**Published:** 2024-07-02

**Authors:** Snježana Kaštelan, Tamara Nikuševa-Martić, Daria Pašalić, Antonela Gverović Antunica, Danijela Mrazovac Zimak

**Affiliations:** 1 Department of Ophthalmology, Clinical Hospital Dubrava, School of Medicine, University of Zagreb, 10000 Zagreb, Croatia; 2 Department of Biology and Genetics, School of Medicine, University of Zagreb, 10000 Zagreb, Croatia; tamara.nikuseva.martic@mef.hr; 3 Department of Medical Chemistry, Biochemistry and Clinical Chemistry, School of Medicine, University of Zagreb, 10000 Zagreb, Croatia; 4 Department of Ophthalmology, General Hospital Dubrovnik, 20000 Dubrovnik, Croatia; agantonela@net.hr; 5 Department of Ophthalmology, University Hospital Centre Zagreb, 10000 Zagreb, Croatia; danijela.mrazovac@gmail.com

**Keywords:** Alzheimer’s disease, age-related macular degeneration, pathophysiology, inflammation, oxidative stress, genetics, epigenetics

## Abstract

Alzheimer’s disease (AD) represents a prominent neurodegenerative disorder (NDD), accounting for the majority of dementia cases worldwide. In addition to memory deficits, individuals with AD also experience alterations in the visual system. As the retina is an extension of the central nervous system (CNS), the loss in retinal ganglion cells manifests clinically as decreased visual acuity, narrowed visual field, and reduced contrast sensitivity. Among the extensively studied retinal disorders, age-related macular degeneration (AMD) shares numerous aging processes and risk factors with NDDs such as cognitive impairment that occurs in AD. Histopathological investigations have revealed similarities in pathological deposits found in the retina and brain of patients with AD and AMD. Cellular aging processes demonstrate similar associations with organelles and signaling pathways in retinal and brain tissues. Despite these similarities, there are distinct genetic backgrounds underlying these diseases. This review comprehensively explores the genetic similarities and differences between AMD and AD. The purpose of this review is to discuss the parallels and differences between AMD and AD in terms of pathophysiology, genetics, and epigenetics.

## 1. Introduction

The global rise in life expectancy brings a greater burden of age-related multifactorial conditions such as neurodegenerative disorders (NDDs) and retinal disorders. NDDs encompass a group of incapacitating and incurable conditions characterized by the gradual deterioration of nerve cells in the brain or peripheral nervous system, resulting in issues with movement or cognitive functions. The leading retinal NDD is age-related macular degeneration (AMD), an acquired and progressive degenerative condition that affects the retina, causing significant impairment of central vision. This impairment results from a combination of non-neovascular factors, such as drusen and abnormalities in the retinal pigment epithelium (RPE), along with neovascular changes including the formation of choroidal neovascular membranes [1]. Likewise, Alzheimer’s disease (AD) represents a chronic NDD, accounting for the majority of dementia cases worldwide [1,2]. The cognitive decline and loss of independence associated with AD among individuals over 65 are exerting significant pressure on families and societies globally, significantly impacting the quality of life and posing considerable challenges to public healthcare. In addition to memory deficits, individuals with AD also experience alterations in the visual system. As the retina is an extension of the central nervous system (CNS), the loss in retinal ganglion cells manifests clinically as decreased visual acuity, narrowed visual field, reduced contrast sensitivity, and difficulties in recognizing visual stimuli [3]. At the pathophysiological level, Aβ plaques and hyperphosphorylated tau deposits (pTau), known as neurofibrillary tangles (NFTs), have been detected in both the retina and the lens. Some researchers correlate this protein accumulation in the retina with analogous aggregations found in the brain. In individuals with AD, a significant reduction in corneal sensitivity has been observed, attributed to nerve damage affecting corneal innervation. This diminished sensitivity is linked to a decline in acetylcholine levels, which is one of the hallmark features of AD [3,4,5]. Diagnosing AD typically involves neuropsychological testing, brain imaging, and/or sampling of cerebrospinal fluid (CSF) [6,7,8]. Neuroimaging techniques while effective, are often costly and not universally accessible. Further, CSF collection is an invasive procedure associated with risks for the patient, necessitating the expertise of well-trained medical professionals. Consequently, research is directed towards alternative diagnostic approaches, such as identifying biomarkers in blood, saliva, and tears, as well as through genetic testing [9,10]. While the disease remains incurable, early recognition of AD is crucial as it enables medical and environmental interventions to slow down symptom progression and enhance the affected individual’s quality of life [11]. 

Like dementia, the prevalence of visual impairment is expected to rise as the population ages [1]. Among the extensively studied retinal disorders, AMD shares numerous aging processes and risk factors with NDDs, such as cognitive impairment which occurs in AD [12,13]. AMD is the leading cause of blindness in older adults, often resulting in irreversible vision loss [14,15]. It is a complex condition marked by progressive degeneration and disruption of the cytoarchitectonics of the central retina. These impairments may arise from drusen formation and alterations in the RPE during the early and intermediate stages of the disease. In advanced stages, AMD may present as central geographic atrophy (non-exudative AMD) or abnormal choroidal neovascularization (CNV), known as exudative AMD [12]. AMD is a prominent factor in the decline of central vision among the elderly. It represents a complex disorder shaped by the interplay of aging, genetic predisposition, and environmental influences [1].

Although the etiology of both conditions is mostly unknown, they exhibit overlapping risk factors, including aging, obesity, atherosclerosis, hypertension, diabetes, smoking, and unhealthy diet, all of which contribute to cellular aging [16,17]. Additionally, they share similar genetic, histologic, and molecular characteristics as well as parallel pathogenic pathways, prompting some researchers to draw comparisons and call AMD ‘Alzheimer’s disease in the eye’ [16]. Given the significant aging of the global population in recent decades, the importance of both diseases has escalated. A comprehensive understanding of their shared mechanisms could offer novel insights into the pathogenesis and treatment of AD and AMD. This review aims to underscore the mutual genetic, histologic, immunologic, and pathogenetic traits of these conditions while assessing aging as a risk factor from this perspective [17].

## 2. Visual Perception and Cognitive Function

Several studies have indicated that visual impairment can serve as an early indicator of abnormal brain aging and AD. These studies have identified shared risk factors and pathogenetic mechanisms including the presence of toxic amyloid oligomers or deposits and related immune modulators [18,19,20,21,22]. Additionally, reductions in the volume of the optic tract [23] and visual cortex, as well as alterations in specific brain networks associated with variations in cognitive abilities, have been observed in AMD patients [24].

In addition to its direct impact on vision, AMD is associated with more pronounced cognitive decline, as evidenced by reduced performance on standardized tests evaluating verbal functioning, processing speed, working memory, visuospatial processing, and attention, compared to individuals without AMD [25,26]. Notably, this decline is particularly evident in verbal fluency, regardless of visual abilities [25,27]. Individuals with AMD, unlike those with normal vision, exhibit differences in brain connectivity within language and memory regions, along with documented regional volume reductions in areas such as the visual cortex, optic radiation, and frontal cortex [28]. 

Limited visual stimulation impacts both the grey matter of the visual and the visual pathways [28,29]. Additionally, cases of central vision loss exhibit cortical thinning and alterations in relaxation parameters within the occipital cortex, with AMD displaying more significant cortical thinning than the localized grey matter changes observed in juvenile macular degeneration [30].

It is still unclear whether AMD pathology directly causes brain changes or whether these changes coincide with AMD due to shared risk factors with brain NDDs, including AD. Recognizing the common characteristics of these NDDs could reveal shared mechanisms and pave the way for new therapies targeting individuals at increased risk of cognitive decline and dementia [24,31].

## 3. Correlation between Alzheimer’s Disease and Age-Related Macular Degeneration 

Although AD and AMD are distinct conditions, they share certain similarities. Both are classified as NDDs and despite affecting different tissues, the fact that the retina is an integral part of the central nervous system suggests a potential correlation between their pathology and pathogenesis [14,17]. Further, several studies have revealed parallels between AD and AMD, including some epidemiological similarities, risk factors, genetic variations, histological and molecular characteristics, and pathogenic mechanisms like oxidative stress, complement activation, and chronic inflammation [32,33].

Histopathological investigations have revealed similarities in pathological deposits found in the retina and brain of patients with AD and AMD [34]. Notably, amyloid β (Aβ), a hallmark component of the detrimental deposits in AD, has also been detected in the drusen of AMD patients, underscoring its potential role in the pathogenesis of both disorders. Moreover, the shared involvement of other pivotal proteins and molecules, including complement factors, has been noted in the pathophysiology of both AD and AMD [34,35]. Additionally, similar abnormalities in miRNA expression profiles have been documented in these two conditions [34,36]. Both diseases exhibit shared molecular features, notably observed in their specific histological features, namely drusen and senile plaques. These include the presence of Aβ, basement membrane proteins, proinflammatory factors, vitronectin, apolipoprotein E, complement components, and inflammatory mediators [17,33,37] (Table 1).

### 3.1. Pathophysiological Characteristics 

Previous epidemiological and clinical studies have revealed associations between AD and AMD, suggesting a potential shared pathogenesis [26,34,38,39,40,41,42,43,44,45,46,47,48]. Nonetheless, certain studies introduce doubts about this connection [49,50,51,52,53] (Table 2). In AMD, early signs include extracellular drusen deposits and basal laminar deposits, containing lipids, glycoproteins, and glycosaminoglycans thought to derive from the degenerating neuroretina. These deposits are associated with the decline in macular function and subsequent loss of photoreceptors. Similarly, In the early stages, AD is characterized by pathological features such as extracellular senile plaques, comprising Aβ, activated microglia, and axons and dendrites from dystrophic neurons. As with AMD, these brain deposits contribute to neuronal damage and cell loss [38].

The formation and composition of drusen serve as a basis for comparing AMD and AD. Drusen are typically located between the RPE and the underlying Bruch’s membrane. While the exact mechanism of drusen formation remains unclear, their presence is associated with RPE atrophy and may result from the accumulation of toxic by-products from the phototransduction cycle. This accumulation leads to inflammation, as well as metabolic and oxidative stress, which play crucial roles in triggering a neovascular response from the underlying choroidal vessels and advancing to neovascular AMD. These deposits can be compared to the formation of senile plaques in the cortex and hippocampus of AD patients, with diffuse plaques appearing in the earlier stages of the disease [33,37].

It IS important to highlight that the substantial overlap between these two diseases is rooted in the shared components found in both the senile plaques of AD and the drusen of AMD, with Aβ particularly prominent [14]. The characteristic pathology of AD entails the progressive accumulation of Aβ plaques and NFT composed of pTau in the brain. Remarkably, Aβ has also been detected in the retina [15] and the retinal pathology observed in AD mirrors the Aβ deposition seen in AMD. Increased deposition of Aβ isoforms has been observed on photoreceptor outer segments and along the RPE–Bruch’s membrane interface in aging human and mouse retinas. Analyses of drusen components have revealed the presence of Aβ within vesicles, particularly in the eyes of patients with advanced AMD. Furthermore, RPE cells have been shown to express amyloid precursor protein (APP) and associated processing enzymes, reacting to Aβ peptides by secreting proinflammatory and proangiogenic factors [33,37]. In addition to Aβ, proteomic analyses have identified other common proteins between drusen and senile plaques, including tau, basement membrane proteins, proinflammatory factors, and components of the complement cascade of the innate immune system [37].

The introduction of advanced imaging technologies like optical coherence tomography (OCT) enables the non-invasive visualization of Aβ accumulation effects on ocular structures in living patients. A comprehensive understanding of the role of amyloid in the eye and brain tissues in these disorders may aid in the discovery of amyloid-based biomarkers as well as the development of more effective therapies for AD and AMD [15].

### 3.2. Chronic Inflammation 

Prolonged inflammation is associated with the onset of various chronic conditions, including autoimmune and NDDs [14]. With age, the levels of amyloid protein and lipofuscin rise in normal eyes and brains, triggering inflammasome, complement system, and autophagy-lysosome function activation, thereby accelerating disease progression [34]. Some research has revealed microglial activation surrounding the drusen and the subretinal space, leading to damage in both the brain and eyes. These occurrences serve as both triggers and outcomes of Aβ formation, establishing an irreversible positive feedback loop involving the secretion of pro-inflammatory cytokines and proteases, which propels disease progression [14]. Furthermore, histological and protein assessments have revealed the presence of complement components, such as active portions of C3, C5, C6–9 (which constitute the membrane attack complex), and regulatory factors B, H, and I, within both drusen and senile plaques [33,37]. These observations imply shared inflammatory pathways in both AMD and AD.

### 3.3. Oxidative Stress 

In both the brain and eyes, cells in physiological conditions require a certain level of oxidative stress to function properly. However, when oxidative stress exceeds a specific threshold, it becomes cytotoxic. The human body has an antioxidant system to restore internal balance, and dysfunction in this system may contribute to the development of AD. It is suggested that oxidative stress could be one of the earliest indicators of AD, as increased levels of heme oxygenase-1, a sensitive marker of oxidative stress, have been detected in the brain tissues of AD patients and individuals with mild cognitive impairment. Similarly, autophagy and lysosomes are involved in the oxidative stress processes in the eyes of patients with AMD and the brains of AD patients. In summary, excessive oxidative stress and dysfunction of mitochondria and lysosomes appear to be common pathophysiological mechanisms in both AMD and AD [14].

Due to their constant activity and turnover, photoreceptors in the macula experience extremely high metabolic and oxidative stress, which increases with age. In such an environment, any deficit in RPE function disrupts the delicate balance, accelerating degeneration. RPE degeneration is further promoted by the accumulation of lipofuscin—cross-linked pigmentary deposits from photoreceptor membranes that RPE cells cannot metabolize. Lipofuscin has harmful oxidant properties and further impairs mitochondrial function. This cycle of metabolic and oxidative stress is worsened by the accumulation of Aβ and other protein aggregates. Many aspects of this mechanism also occur in the brain of patients with AD, including increased reactive oxygen species (ROS) and oxidative damage, lipofuscin formation, and mitochondrial dysfunction [33,37].

Oxidative stress plays a significant role in the development and progression of AMD, with antioxidants being crucial in mitigating this damage. Poor dietary habits can lead to nutrient deficiencies, which are associated with the advancement of AMD. The use of antioxidant supplements, particularly a combination of vitamin C, vitamin E, β-carotene, zinc, and copper, has been shown to reduce the risk of progression from early to late AMD. Additionally, adherence to a Mediterranean diet rich in antioxidants has been found to lower the risk [54].

Hyperglycemia in diabetic patients disrupts retinal homeostasis by inducing inflammatory responses in cells and oxidative stress. Although no clear association has been established between diabetes and early AMD, research indicates that diabetes is a risk factor, particularly for the late stage. The connection between diabetes and AMD might be mediated through several biological pathways, particularly those involving inflammation suggesting that inflammation plays a significant role in linking diabetes with AMD. Chen et al. found that advanced glycation end products (AGEs), which are more prevalent in diabetic individuals, could contribute to the progression of AMD. AGEs and their receptors are known to colocalize in AMD, indicating that these glycation products may be involved in the disease mechanism [55]. 

## 4. The Role of Susceptibility Genes in Age-Related Macular Degeneration and Alzheimer’s Pathogenesis

The role of susceptibility genes in both AMD and AD pathogenesis underscores the complex interplay between genetic factors and disease development.

AD and AMD exhibit numerous parallels in their clinical and pathological characteristics, notably in response to stress factors like oxidative stress and inflammation. Both conditions manifest detrimental intra- and extracellular deposits with comparable features. Cellular aging processes demonstrate similar associations with organelles and signaling pathways in retinal and brain tissues. Despite these similarities, there are distinct genetic backgrounds underlying these diseases. This review comprehensively explores the genetic similarities and differences between AD and AMD.

In AMD, several susceptibility genes have been identified, with genes that are involved in the regulation of the immune response and inflammation suggesting that dysregulation of these processes may contribute to AMD pathogenesis. Similarly, in Alzheimer’s disease, susceptibility genes play a critical role in disease risk. The genes associated with AD risk include genes that are involved in various biological pathways, including lipid metabolism, immune response, and neuronal function, highlighting the multifactorial nature of AD pathogenesis.

Overall, the identification of susceptibility genes in both AMD and AD has provided valuable insights into the underlying mechanisms of these diseases. However, further research is needed to fully understand how genetic variants contribute to disease development and to develop targeted therapies for these devastating conditions.

Research indicates that individuals with a sibling or a parent affected by AMD are 12–27 times more likely to develop AMD compared to individuals in the general population [56].

In the International age-related macular degeneration genomics consortium study, which included 43,566 participants primarily of European descent, consisting of 17,832 controls, 16,144 advanced AMD cases, and 6657 individuals with intermediate AMD, researchers identified 52 independently associated variants across 34 loci, all showing significant genome-wide associations (*p* < 5.0 × 10^−8^). The genetic landscape of AMD is intricate, with 52 distinct variants identified across 34 genetic loci, as revealed by the most extensive genome-wide association study conducted to date [57]. Given that over 50% of the heritability of AMD can be attributed to two primary loci containing variations in both coding and non-coding regions on chromosomes 1q (CFH) and 10q (ARMS2/HTRA1), AMD stands out as one of the most thoroughly genetically characterized complex disorders [58,59]. Many studies agree on the relationship between the inflammatory system, chemokines, and their corresponding ligands in AMD [60]. Particularly notable was the discovery that ten variants situated in seven extracellular matrix genes like COL15A1, COL8A1, MMP9, PCOLCE, MMP19, CTRB1-CTRB2, and ITGA7 were associated solely with advanced AMD (neovascular AMD and geographic atrophic AMD [57].

The Complement Factor H (CFH) gene plays a crucial role in activating C3 cleavage and facilitating the degradation of its products. The rs1061170 CFH genetic variant has been strongly associated with AMD, as it diminishes the binding affinity to heparan sulfate proteoglycans, leading to lipoprotein accumulation in Bruch’s membrane and subsequent progression of drusen [61]. The CFH H402 variant exhibits reduced MDA-binding efficiency, thus compromising the binding of oxidized phospholipids. This interplay between oxidative stress, lipid metabolism, and complement dysregulation underscores its role in AMD pathogenesis. These alterations in CFH function diminish its ability to regulate the complement pathway effectively [62]. This reduced suppression of complement activation elevates MAC levels, contributing to retinal cell damage and impairing debris removal, ultimately fostering the accumulation of waste products in drusen [63]. Sharma et al. found significantly lower serum CFH levels in individuals with the AMD-associated Y402H variant compared to controls, suggesting its potential as a disease biomarker [64]. The rs1061170/Tyr402His variant is closely linked with rs570618 and another significant variant, rs10922109. Moreover, rare CFH mutations have been identified, presumed to confer distinct disease phenotypes and earlier disease onset. Among these, rs121913059 shows increased risk independently of common variants. Additionally, the CFHI62V polymorphism represents a protective haplotype [65].

Research suggests that polymorphisms within the extended Complement Factor H (CFH) locus could potentially influence the Complement-Factor-H-Related (CFHR) genes. Although the precise role of CFHR genes remains largely unexplored, they are hypothesized to be involved in complement regulation, potentially by competing with Factor H [66].

The relationship between rs2230199 and rs1047286 variants within the C3 gene and susceptibility to AMD has been frequently documented [67]. Furthermore, a total of 71 rare missense variants have been identified, with some showing a strong association with AMD [68].

CFB plays a regulatory role in the alternative complement pathway, similar to CFH. Overall, certain rare alleles in CFB may dampen the activation of the alternative complement pathway, thus exerting a protective role against the onset of AMD [69].

A rare missense variant in the C9 gene, known as rs34882957, has been linked to AMD [70].

The CFI gene encodes a serine proteinase vital for complement regulation [71]. Carriers of the rs2285714 variant have been strongly linked to an increased risk of AMD [72], suggesting that this mutation could potentially serve as a biomarker [73].

SERPING1 encodes C1 inhibitor, a member of the serine proteinase inhibitor superfamily that primarily regulates the complement system by inhibiting spontaneous activation. It is expressed in the neural retina, RPE, and choroidal tissue, and has been observed to contribute to the predisposition to AMD in Caucasian populations [74].

Toll-like receptor 3 (TLR3) is a key gene in the intricate inflammatory cascade, responsible for initiating programmed cell death in virus-infected cells through the recognition of double-stranded RNA. Additionally, TLR3 is implicated in the neo-angiogenesis process, as indicated by its increased expression during choroidal neovascularization (CNV) formation. Variants that impair or diminish these functions of TLR3 may contribute to the pathobiology of various subtypes of AMD [75].

In addition to CFH, PLEKHA/ARMS2/HTRA represents a major and prevalent genetic risk factor for AMD. The rs10490924/A69S polymorphism, located near the ARMS2 and HTRA1 genes, has been identified as a robust genetic risk factor for AMD [76]. The ARMS2 A69S polymorphism has been shown to influence the response to anti-VEGF treatment in late-stage AMD, particularly in the East Asian population [77]. This suggests that detecting this mutation, especially in A-allele carriers or those with the AA genotype, could serve as a prognostic indicator for the anti-VEGF response and may warrant early intervention [78]. Furthermore, other studies have highlighted the significance of the regulatory region HTRA1 rs11200638. HTRA1 encodes a heat shock serine protease that regulates the cleavage of extracellular matrix (ECM) proteoglycans, with its overexpression leading to increased remodeling of the Bruch’s membrane. This disruption of ECM structural integrity renders the choriocapillaris more susceptible to neovascular invasion, a hallmark of wet AMD. Moreover, HTRA1 counteracts the activity of transforming growth factor-β (TGF-β), a key player in ECM deposition and neo-angiogenesis. Taken together, these findings suggest that the deleterious effects of HTRA1 may contribute to the invasion of choroidal neovascularization (CNV). These common variants are in strong linkage disequilibrium (LD) with rs3750486, the first SNP identified at the locus [69].

The retina is highly susceptible to oxidative stress, which is a primary cause of DNA damage. The manganese superoxide dismutase (MnSOD) enzyme, located in the mitochondrial matrix, plays a critical role in cellular defense against reactive oxygen species (ROS). MnSOD functions by converting superoxide radicals into hydrogen peroxide, thus mitigating oxidative damage. There are four known functional polymorphism sites for MnSOD, among which the Ala-9Val and Ile58Thr variants have been particularly studied. Research has shown a significant association between these MnSOD gene polymorphisms and the development of wet AMD. Specifically, a more efficient form of the MnSOD enzyme resulting from these polymorphisms may lead to an excess production of hydrogen peroxide. This surplus hydrogen peroxide can further react with ferrous iron to generate highly reactive hydroxy radicals, thereby increasing the overall ROS burden within cells and contributing to the pathogenesis of AMD [79].

Iron serves as a source of reactive oxygen species (ROS) generation through processes such as the Haber–Weiss and Fenton reactions, leading to cellular damage. Studies have shown that the levels of iron are notably elevated in eyes affected by AMD compared to healthy eyes. This suggests that iron accumulation may contribute to the pathogenesis of AMD [80]. Furthermore, enzymes like heme oxygenase-1 (HO-1) and heme oxygenase-2 (HO-2), encoded by the HMOX1 and HMOX2 genes, respectively, are important regulators of iron-induced oxidative stress. Polymorphisms in these genes, such as the HMOX1 rs2071747 variant associated with the progression of AMD to the wet form, and the HMOX2 rs2270363 variant linked to dry AMD, further underscore the role of iron metabolism in AMD development [81].

Apolipoprotein E (ApoE) plays a crucial role in cholesterol transport and regulating its availability to cells by facilitating the interaction between lipoproteins and LDL receptors. It is found in various ocular tissues, including photoreceptor segments, the retinal ganglion layer, and Bruch’s membrane. Studies have consistently shown that the ε2 allele of ApoE is associated with an increased risk of advanced AMD, whereas the ε4 allele appears to have a protective effect against wet AMD [82].

Mutations in the ABCA4 gene are primarily associated with Stargardt disease, a form of inherited macular degeneration that affects the retina. However, it is important to note that variants in the ABCA4 gene can lead to a spectrum of phenotypical manifestations, one of which includes age-related macular degeneration (AMD). This highlights the genetic complexity and heterogeneity of AMD, with ABCA4 mutations being just one of the contributing factors to the development of the disease. 

Loss of function mutations in the ABCA4 gene leads to the accumulation of N-retinylidene phosphatidylethanolamine (N-retinylidene PE) complexes, resulting in an increased load of lipofuscin within RPE cells. This accumulation disrupts cholesterol metabolism in the RPE cells, leading to uncontrolled cholesterol levels. ABCA1, on the other hand, is a membrane transporter responsible for removing excess cholesterol from cells and transferring it to lipid-poor apolipoprotein A-I (Apo A-I), a major component of high-density lipoproteins (HDLs). Mutations in ABCA1 cause a significant decrease in HDL levels, leading to cholesterol build-up in macrophages and an increased risk of cardiovascular disease.

Specific genetic variants in ABCA4 have been associated with the risk of developing AMD. For example, the variant rs1883025 is linked to a decreased risk of AMD development, while rs2740488 is associated with an increased risk. These findings highlight the role of ABCA4 gene variants in modulating the risk of AMD and further underscore the intricate interplay between lipid metabolism and AMD pathogenesis [83].

Hepatic Lipase (LIPC) is abundantly expressed in the retina and RPE, where it plays a role in regulating serum levels of high-density lipoprotein (HDL) cholesterol. The variant rs2043085 is associated with an increased risk of advanced AMD. These genetic variations in the HDL pathway suggest their potential utility as biomarkers for AMD susceptibility [57].

To maintain DNA integrity, efficient DNA repair mechanisms are crucial [84]. Polymorphisms in genes encoding uracil-DNA glycosylases (UDGs), such as SMUG1 and UNG2, have been proposed to be involved in AMD pathogenesis. Additionally, the XPD gene, which encodes a DNA helicase involved in transcription, nucleotide excision repair (NER), and apoptosis, may also play a role. Variants in the XPD gene could potentially affect the efficiency of NER, leading to DNA alterations and contributing to AMD development [85].

Fibuline 3 (EFEMP1) and Fibuline 5 (FBLN5) are members of the matrix glycoprotein family, characterized by multiple EGF-like sequences. EFEMP1 is known to induce the release of TIMP-1 and TIMP-3 and neutralize various extracellular matrix metalloproteinases [86]. Mutations in EFEMP1 are associated with Doyne honeycomb retinal dystrophy, a maculopathy sharing common features with AMD. In AMD-affected maculae, misfolded EFEMP1 accumulates between the RPE layer and drusen. Although it is not a constituent of drusen, its presence may contribute to its formation and subsequent macular damage. Fibuline 6, also known as Hemicentin (HMCN1), belongs to the extracellular immunoglobulin (Ig) superfamily and shares structural similarities with EFEMP1, including EGF-like repeats. HMCN1 is one of the few genes implicated in the inheritance of AMD [87].

Genetic tests currently utilize variations in CFH and ARMS/HTRA1 genes. However, the genetic architecture not only influences the risk of developing AMD but also affects the response to therapeutic interventions [88]. The most important genes and related polymorphisms are presented in Table 3.

Two forms of AD have been identified: familial and sporadic. Familial AD typically presents as autosomal dominant with early onset (EOAD), affecting individuals under 65 years old, which accounts for 1–5% of cases [89]. It is characterized by specific genetic alterations, including mutations in genes such as presenilin 1 (PSEN1) located on chromosome 14q24.2, which is identified in up to 70% of familial AD cases, presenilin 2 (PSEN2) on chromosome 1q42.13, and the APP gene on chromosome 21q21.3. [90].

On the other hand, sporadic AD presents as late-onset (LOAD) and typically occurs in individuals aged 65 years and older. While aging is considered the primary risk factor for sporadic AD, other risk factors have been identified. These include female sex, traumatic brain injury, depression, environmental pollution, physical inactivity, social isolation, low academic level, metabolic syndrome, and genetic susceptibility, particularly mutations in the ε4 allele of apolipoprotein E (APOE) located on chromosome 19q13.32. Sporadic AD is a complex disorder with a heritability estimated to be as high as 60–80% [91].

According to the amyloid hypothesis, numerous genes implicated in pathological Aβ processing have been identified. For instance, disintegrin and metalloproteinase domain-containing protein 10 (ADAM10), the primary α-secretase in the brain, may lead to alterations in the Aβ42/Aβ40 ratio [92]. Similarly, phosphatidylinositol-binding clathrin assembly protein (PICALM) facilitates clathrin polymerization, contributing to the formation of neuritic plaques [35,36,37]. Additionally, clustered mitochondria protein homolog (CLU), acting as a chaperone to prevent the aggregation of foreign proteins, has been shown to inhibit amyloid fibril formation by APP and other associated genes.

However, pathological Aβ processing alone does not fully elucidate the complexity of AD. In light of this, the neuroimmunomodulation hypothesis has been proposed, suggesting that AD arises from the response of glial cells to damage signals, triggering a neuroinflammatory cascade and subsequent dysregulation of protein kinases and phosphatases that promote hyperphosphorylation and oligomerization of the Tau protein. These Tau oligomers and filaments released post-neuronal apoptosis can reactivate microglia, perpetuating a detrimental signaling cycle underlying the neurodegeneration observed in AD and other tauopathies [93]. Supporting this hypothesis, numerous genes implicated in the immune response have been identified. For example, the transcription factor PU.1 (SPI1), which regulates immune-related genes in myeloid cells, may contribute to AD by modulating key immune pathways and altering the epigenetic landscape [94]. Additionally, the myeloid cell surface antigen CD33, involved in the negative regulation of cytokine production, could influence microglial activation and, consequently, AD pathology [95].

Furthermore, genes involved in Tau metabolism have also been linked to the neuroimmunomodulation hypothesis. Bridging integrator 1 (BIN1), for instance, participates in membrane tubulation and interacts with microtubule-associated proteins like Tau, in addition to its roles in endocytosis and intracellular endosome trafficking [96]. Triggering receptor expressed on myeloid cells 2 (TREM2) has been associated with CSF Tau levels [97]. Similarly, loss of function of scaffolding CD2-associated protein (CD2AP), involved in intracellular trafficking and cytoskeletal organization, has been implicated in Tau-induced neurotoxicity, aberrant neurite structure modulation, and blood–brain barrier disruption [98].

The infectious hypothesis posits that pathogens like viruses, bacteria, or prions can trigger an inflammatory response, leading to the aggregation of Tau and Aβ proteins, which further exacerbates inflammation [99]. In this context, the paired immunoglobulin-like type 2 receptor alpha (PILRA), an inhibitory immunoglobulin receptor that regulates the immune system and serves as a co-receptor for herpes simplex virus type 1 (HSV-1), has been implicated in microglial activation dysfunction, potentially linking infectious agents to AD pathogenesis [100]. Additionally, APO-ε4 has been associated with HSV-1 infections [101]. This connection between infectious diseases and AD underscores the significance of considering infectious factors in both diagnostic and therapeutic approaches.

Moreover, beyond the pathological processing of Tau and Aβ, other neuropathological events may be triggered by genes associated with metabolic disorders, particularly those involved in lipid/atherosclerosis pathways and insulin resistance [102]. For instance, solute carrier family 10 member 2 (SLC10A2) plays a crucial role in regulating cholesterol metabolism. Accumulation of cholesterol in neurons has been linked to neuronal death, memory impairment, and increased Aβ production [103]. Given that AD has been characterized as a metabolic disease influenced in part by insulin resistance, zinc finger CW-type PWWP domain protein 1 (ZCWPW1), which is involved in positively regulating DNA metabolism, may reduce the risk of late-onset AD by suppressing insulin resistance [104]. The main genes involved in the pathophysiology of AD are presented in Table 4.

**Figure 1:**
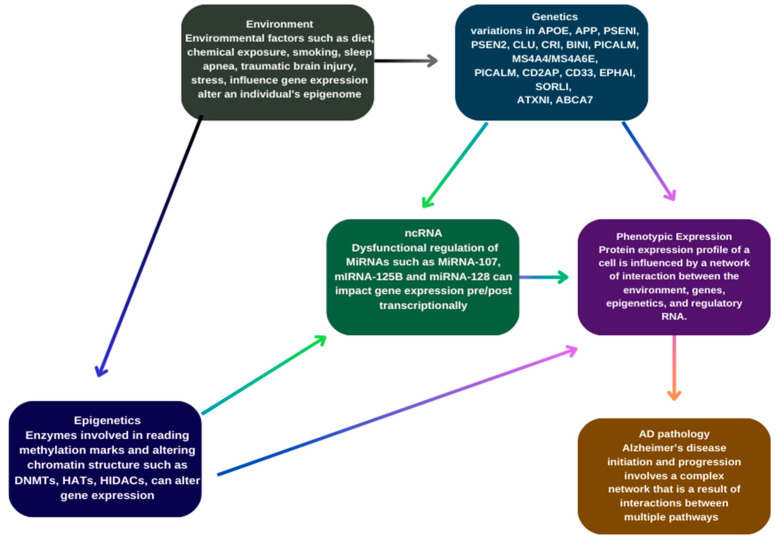
Genetic and epigenetic factors contributing to Alzheimer’s disease.

Additionally, genes associated with the AMP-activated protein kinase (AMPK) pathway have been linked to AD risk through their roles in regulating energy balance, glucose and lipid metabolism [63], autophagy dysfunction leading to Aβ and Tau pathology and altering synaptic plasticity in hippocampal neurons [105]. Similarly, methylenetetrahydrofolate reductase (MTHFR), a rate-limiting enzyme in the methyl cycle, may be implicated as high levels of homocysteine, observed in AD patients, are associated with vascular damage, increased inflammation, and endothelial dysfunction [106]. Figure 1 represents different factors contributing to AD.

## 5. Role of Epigenetic Mechanisms in Age-Related Macular Degeneration and Alzheimer’s Pathogenesis

**Figure 2:**
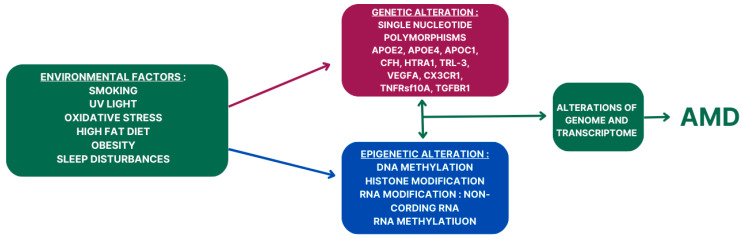
The role of interplays of genetic and epigenetic factors in AMD pathogenesis.

The role of epigenetic mechanisms in AMD and AD pathogenesis is an emerging area of research that holds significant promise for understanding these complex diseases. In AMD, epigenetic alterations have been implicated in the dysregulation of gene expression in the retinal pigment epithelium (RPE) and choroid, contributing to the pathological changes observed in the disease. DNA methylation, histone modifications, and noncoding RNAs have all been implicated in AMD pathogenesis. For example, aberrant DNA methylation patterns in AMD-affected tissues have been associated with the dysregulation of genes involved in inflammation, oxidative stress, and angiogenesis, processes that are critical in AMD pathophysiology. Similarly, alterations in histone modifications have been linked to changes in gene expression in AMD, further highlighting the role of epigenetic regulation in disease progression. A schematic overview of genetic and epigenetic factors in AMD pathogenesis is presented in Figure 2.

In AD, epigenetic mechanisms have also been implicated in disease pathogenesis. DNA methylation patterns, histone modifications, and microRNA dysregulation have all been observed in the brains of AD patients. These epigenetic alterations can impact the expression of genes involved in neuroinflammation, synaptic function, and amyloid processing, key processes implicated in AD pathophysiology. 

Overall, the role of epigenetic mechanisms in AMD and AD pathogenesis is complex and multifaceted. Understanding how epigenetic alterations contribute to disease progression may provide new insights into disease mechanisms and identify novel therapeutic targets for these devastating neurodegenerative conditions. Further research in this area is warranted to elucidate the precise role of epigenetic mechanisms in AMD and AD and to develop targeted therapies aimed at modulating these processes.

The term has evolved to include any process that alters gene activity without changing the DNA sequence and leads to modifications that can be transmitted to daughter cells. DNA methylation, histone modifications, and noncoding RNAs stand as the focal points of extensive research within the realm of epigenetic mechanisms. Hence, this shift prompts changes in the transcriptional activity of genes implicated in the development of NDDs. Over recent years, it has become increasingly evident that epigenetic mechanisms play a significant role in the onset and progression of AD and AMD.

To explore the potential impact of epigenetic mechanisms on antioxidant genes relevant to AMD, Hunter et al. [107] conducted a study assessing DNA methylation and its effect on gene expression. Glutathione S-transferase PI (GSTP1) plays a critical role as a scavenger of reactive oxygen species (ROS), offering protection against genome-damaging oxidants. The absence of GSTP1 could lead to reduced defense against oxidative insults, rendering individuals more susceptible to oxidative stress.

Hunter et al. [107] observed a significant reduction in mRNA levels of glutathione S-transferase isoforms mu1 (GSTM1) and mu5 (GSTM5) in AMD patients compared to age-matched controls, particularly in the retinal pigment epithelium (RPE)/choroid and neurosensory retina. This reduction correlated with hypermethylation of the GSTM5 promoter region. Interestingly, mRNA and protein levels were more prominently decreased in the RPE compared to the neurosensory retina in post-mortem AMD samples, irrespective of age.

Their findings suggest that a comparison of DNA methylation patterns, along with mRNA levels, revealed notable differences between AMD and normal retinas. This indicates that GSTM1 and GSTM5 may undergo epigenetic repression in AMD RPE/choroid, potentially heightening susceptibility to oxidative stress in AMD retinas [107]. Oxidative damage in retinal pigment epithelial (RPE) cells results in decreased mRNA and protein levels of detoxification enzymes, including glutathione S-transferase (GSTM1 and GSTM5), due to hypermethylation of the GSTM1 promoter. This hypermethylation leads to reduced expression of GSTM1 and GSTM5 at both the mRNA and protein levels in the RPE and choroid, rendering patients with AMD more susceptible to oxidative stress. Moreover, both mitochondrial and nuclear DNA extracted from RPE cells of AMD patients exhibit increased oxidative damage, indicating the involvement of imbalanced redox enzymes in AMD pathogenesis [108].

Wei et al. investigated the role of methylation changes in the interleukin 17 receptor C (IL17RC) gene, an essential component of the IL-17 receptor complex involved in mediating proinflammatory activities. Initially, they examined genome-wide differences in DNA methylation patterns between twins with discordant AMD and validated methylation changes at the IL17RC promoter in discordant siblings as well as in an AMD case-control cohort. They also evaluated IL17RC expression in the eyes and blood of AMD patients. Their findings revealed a significant decrease in methylation levels on the IL17RC promoter in AMD patients. Moreover, they demonstrated that hypomethylation of the IL17RC promoter correlated with increased expression of its protein and mRNA in peripheral blood as well as in the affected retina and choroid [109]. This suggests that DNA methylation patterns and IL17RC expression could potentially serve as biomarkers for AMD diagnosis and may play a role in disease pathogenesis. In contrast, Oliver et al. did not find evidence of differential methylation between AMD cases and age-matched controls in their study. They concluded that hypomethylation within the IL17RC gene promoter in peripheral blood may not be suitable as a clinical biomarker for AMD [110].

Nucleosomes contain highly alkaline, positively charged amine groups that enable histone proteins to interact with and bind to the negatively charged phosphate backbone of DNA. During acetylation, these amine groups on the histone molecules are converted into amides, neutralizing the positive charges on the histones. As a result, the histones’ ability to bind to DNA is reduced, leading to the expansion of chromatin and allowing for genetic transcription. Conversely, histone deacetylation, catalyzed by histone deacetylases, removes acetyl groups from histones, increasing the presence of positively charged amine groups. This facilitates high-affinity binding between the histones and the phosphate backbone of DNA [111].

Hypoxia-inducible factor-1α (HIF1α), known for regulating genes like VEGF, is implicated in the development of neovascular AMD. Histone deacetylase inhibitor (HDACi) reduces HIF1α and VEGF expression. Hypoxia boosts histone lysine demethylase (KDM) activity in RPE cells, promoting pro-angiogenic gene expression. HDAC7 reduction impedes HIF1α transactivation, yet VEGF induces HDAC7 nuclear egress, activating angiogenic gene expression. HDACi like Trichostatin A (TSA) inhibit HDACs, endothelial cell proliferation, and VEGF-receptor expression [112].

Another significant aspect in the investigation of histone acetylation/deacetylation in relation to AMD involves SIRT1, which encodes NAD-dependent deacetylase sirtuin-1, a crucial enzyme in histone deacetylation [113]. SIRT1 regulates various cellular processes including cell senescence, DNA damage repair, apoptosis, inflammation, and angiogenesis. SIRT1 plays a pivotal role in maintaining cellular homeostasis, and dysregulation of its expression may contribute to pathological conditions. 

Indeed, SIRT1 plays a protective role in RPE cells by safeguarding them from cell senescence and apoptosis triggered by oxidative stress. SIRT1 expression was notably diminished in RPE cells from AMD patients compared to age-matched controls [114]. However, contrasting findings showed heightened SIRT1 expression in human CNV membranes compared to non-AMD donor eyes [115].

Epigenetic changes, including disruptions in enzyme activity responsible for DNA methylation and histone modifications, contribute to the pathology. These genetic and epigenetic modifications induce alterations in phenotypic expressions, ultimately contributing to the development of Alzheimer’s disease [116]. In Alzheimer’s disease cases, aberrant DNA methylation and DNA hydroxymethylation occur in specific regions of the genome, primarily due to abnormal epigenetic mechanisms affecting CpG islands, thus initiating pathological alterations. In Alzheimer’s disease cases, DNA methylation and DNA hydroxymethylation are seen in certain regions of the genome; this is due to the abnormal epigenetic mechanism of CpG island initiating the pathologic alteration in Alzheimer’s disease [117]. Highlighted that there is an increase in DNA hydroxymethylation levels in Alzheimer’s disease subjects compared to age-matched controls. DNA methylation in the promoter region decreases the extended regions of cytosine and guanine repeats in the mammalian genes. These sites are heavily targeted by DNMTs and are known to modulate gene expression [118].

The SH-SY5Y neuronal-like cell lines, when treated with conditioned media, exhibited a mutation known as Indiana (V717F) alongside higher concentrations of Aβ peptides [119]. However, DNA microarray analysis of IMR-32 neuroblastoma cell lines treated with elevated levels of synthetic Aβ peptides did not reveal significant DNA methylation alterations [120]. Conversely, studies using antibodies recognizing methylated DNA observed DNA methylation in brain regions such as the entorhinal cortex and hippocampus of Alzheimer’s patients, while other studies employing the same method reported no differences in regions like the entorhinal cortex, frontal cortex, temporal cortex, and hippocampus [121]. Furthermore, hypomethylation within specific promoter regions of the CRTC1 gene was observed to decrease in the human hippocampus of Alzheimer’s-affected individuals compared to controls. Methylation within prom1 demonstrated a notable inverse correlation with p-tau deposition [122].

In the J20 mouse model, an age-related decrease in DNA methylation was found in the dentate gyrus, and a decrease in the ratio between DNA methylation and hydroxymethylation was also found in the dentate gyrus and cornu ammonis 3. Only the J20 model showed an age-related reduction in global DNA methylation, while DNA hypermethylation was observed in the 3xTg–Alzheimer’s disease model [23]. Studies have indicated an elevation in DNA hydroxymethylation levels in Alzheimer’s subjects compared to age-matched controls. DNA methylation in the promoter region leads to decreased cytosine and guanine repeats in extended genomic regions, which are crucial sites targeted by DNMTs known to regulate gene expression. In the J20 mouse model, an age-related decline in DNA methylation was observed in the dentate gyrus, accompanied by a reduction in the ratio between DNA methylation and hydroxymethylation in both the dentate gyrus and cornu ammonis 3. Notably, only the J20 model displayed an age-related decrease in global DNA methylation, while the 3xTg–Alzheimer’s disease model exhibited DNA hypermethylation [123].

In general, histone acetylation, particularly at H3 and H4 lysine residues, is associated with enhanced transcriptional activity, whereas histone deacetylation and methylation tend to inhibit gene expression [124]. However, compared to research on DNA methylation, studies on histone modifications, especially in the context of Alzheimer’s disease, are relatively scarce.

Guan et al. investigated histone deacetylase expression in mice and found that overexpression of histone deacetylase 2 led to a decrease in dendritic spine density, synapse number, and memory [125]. Moreover, elevated levels of histone deacetylase 6, known to modulate tau phosphorylation and accumulation, were observed in brain regions such as the cerebral cortex and hippocampus tissues of Alzheimer’s disease patients compared to control subjects [126]. Additionally, heightened levels of phosphorylated H2AX at Ser139 were detected in hippocampal astrocytes of individuals with Alzheimer’s disease [127].

Gupta et al. [128] observed that inhibiting histone deacetylase with sodium butyrate led to increased levels of H3K4 trimethylation and decreased levels of H3K9 demethylation in the hippocampal regions of the brain. Their in vivo and in vitro studies, along with investigations in Alzheimer’s subjects, revealed a notable upregulation of histone deacetylase II associated with the transcriptional activation of memory and learning-related genes. Modulating the activity of histone deacetylase II demonstrated potential in rejuvenating neuronal and synaptic structures, thereby ameliorating cognitive deficits. Additionally, genome-wide analysis indicated distinctive patterns of lysine H3K27 acetylation (H3K27ac) in Alzheimer’s subjects, particularly in the entorhinal cortex, showcasing pronounced marks of active enhancers and promoters correlated with gene expression and transcription factor binding. These findings were elucidated through chromatin immunoprecipitation followed by highly parallel sequencing [129].

Zhang et al. [130] conducted an investigation revealing decreased levels of histone acetylation in the temporal lobes of the brains of Alzheimer’s subjects compared to aged controls. Conversely, Naryan et al. [131], in their study examining histone acetylation levels in postmortem brains of Alzheimer’s disease patients, concluded that acetylation levels were elevated in the brains of individuals with Alzheimer’s. The influence of Aβ peptide excess on histone regulation was noted. In Tg2576 mice, increased histone H3 acetylation and phosphorylation were observed in the prefrontal cortex, alongside elevated histone H3 methylation in the same region but reduced levels in the striatum. Additionally, higher levels of histone H4 acetylation were noted in the CA1 region of the hippocampus [132].

During the hydrolysis of APP into Aβ peptide, an additional product known as the APP intracellular C-terminal domain (AICD) is generated [133]. AICD, along with Fe65 and Tip60 (HAT), forms trimeric complexes that regulate histone acetylation-related genes. These complexes synchronize Alzheimer’s disease-related genes, including APP, glycogen synthase kinase 3 beta, β-site of APP cleaving enzyme 1, and neprilysin. The AICD-Fe65 complex plays a crucial role in managing APP. However, overexpression of Fe65 can lead to increased production of Aβ peptides, thus promoting the onset and progression of AD [134]. An overview of epigenetic factors involved in the pathogenesis of AMD and AD is presented in Table 5.

### Similarities in miRNA Profiling

Both AMD and AD share several pathogenetic mechanisms involving mRNA and miRNA. These entail similar pathological signaling abnormalities and illness mechanisms at the molecular genetic level that cause degeneration of retinal and neural cells as well as inflammatory neuropathology.

Certain miRNAs appear to be significant factors in genetic regulatory mechanisms related to inflammatory neurodegenerative pathways, which are present in AD and AMD. The degenerating neocortex and the retina share five of the top 12 changed miRNAs [135]. In 2012, Lukiw et al. [135] reported that age-related degeneration of the brain and retina is associated with the upregulation of four miRNAs: miRNA-9, miRNA-125b, miRNA-146a, and miRNA-155. Since these four miRNAs specifically target the complement factor H (CFH) mRNA 3′UTR and downregulate CFH expression, it has been demonstrated that they are implicated in the upregulation of inflammatory signaling [135]. In a study conducted on the pooled neocortical and retinal tissues, there was a common and significant upregulation of several miRNAs listed in the typical miRNA array profiles for AMD retinal (pooled macular) and AD (pooled temporal lobe neocortex). These miRNAs included miRNA-7 and the Let-7 cluster, miRNA-23a and the miRNA-27a cluster, miRNA-9, miRNA-34a, miRNA-125b, miRNA-155, and miRNA-146a [136]. Serum miRNA profiling of patients with AMD and AD showed dysregulation of miR-9, miR-23a, miR-27a, miR-34a, miR-146a, and miR-155 [36]. 

MiRNA miRNA-9, miRNA-125b, miRNA-146a, and miRNA-155 were present in all the investigations mentioned above. This suggests that AMD and AD share a similar pathophysiological background. 

In the growing brain, the role of mir-9 in neurogenesis and angiogenesis has been documented. It has been discovered that the suppression of mir-9 causes alterations in the transcriptional factors TLX and ONECUT to express at higher levels, which in turn causes VEGF-A overexpression [137]. TLX is essential for adult neurogenesis and brain growth while ONECUT components are necessary for the proper development of motor neurons.

In both developing and terminally differentiated RPE cells, the let-7 family of miRNAs and miR-125b-5p were reported to be among the most abundantly expressed miRNAs [138]. Functional investigations demonstrated that miR-125b-5p and let-7 promoted RPE cell development and produced a more robust RPE state.

## 6. Future Perspectives 

AD and AMD represent prevalent and incapacitating age-related conditions. Worldwide, over 43 million individuals are affected by dementia, with AD being the most prevalent form, while an estimated 170 million people worldwide contend with AMD. Unfortunately, effective therapeutic interventions for both AD and AMD are currently elusive. Presently, only symptomatic treatments are available for AD, offering limited help. Similarly, although the introduction of anti-angiogenic therapy has been beneficial in preventing blindness and restoring vision in cases of exudative or wet AMD [139], recent investigations have identified potential shared connections between AD and AMD, offering new insights into their pathogenesis, genetic basis, development of innovative biomarkers, and personalized treatment approaches for both conditions [15].

Aging emerges as a significant risk factor for both AD and AMD [24] and numerous studies have demonstrated a link between aging and the development of these conditions [38,48,49]. Specifically, both exhibit histopathological accumulation of Aβ, accompanied by oxidative stress and local inflammation preceding clinical symptoms [24]. While there is still limited genetic or epidemiological evidence linking AD and AMD, these neurodegenerative conditions share many risk factors. They may represent distinct yet interconnected amyloidopathies, suggesting a potential benefit from targeted therapeutic approaches based on similar principles. This review of the pathophysiological and genetic aspects of AD and AMD aimed to highlight their shared features and highlight potential susceptibility biomarkers. 

In general, numerous genetic variations have a significant and intricate influence on disease progression, manifesting multiple effects on pathogenesis pathways. Through the integration and modeling of diverse genetic and genomic datasets, some genetic associations contributing to the pathogenesis of both diseases have been identified [34]. 

Regarding the risk of AMD across diverse populations worldwide, research suggests that approximately 34 genetic loci may contribute to between 47% and 71% of genetic susceptibility, while non-genetic factors are estimated to explain 19% to 37% of the disease risk [12]. Given some of their connections, unraveling the genetic background of AMD could shed light on some of the molecular components and pathways implicated in the pathophysiology and susceptibility of AD. Investigating genetic determinants linked to AMD could therefore offer insights into some of its associations with AD, having potential future clinical implications. Special focus should be placed on non-coding variants, as they can influence regulatory genetic and epigenetic networks that are relevant to both AMD and AD [14].

The identification of susceptibility genes in prior research highlights the impact of population-specific genomic variations on disease onset and progression. These insights aid in comprehending the varying prevalence of AD and AMD among different populations and underscore the multifactorial nature of both conditions, characterized by a combination of genetic, environmental, and lifestyle factors. Environmental influences, subject to change over time, contribute to the selection of specific genetic variants and their influence on susceptibility to multifactorial diseases, including AD and AMD [13,14]. Some previous studies suggesting a common genetic background influencing susceptibility to both AD and AMD propose that individuals with AMD may face an elevated risk of developing AD during their lifetime. Other research indicates that the presence of dementia and AD increases the likelihood of developing AMD, implying a potential bidirectional relationship [26,34,38,39,40,41,42,43,44,45,46,47,48]. These findings suggest that AMD could potentially serve as a marker for AD, facilitating early diagnosis and enabling treatment to mitigate or slow cognitive decline [44]. However, it should be noted that not all previous studies have confirmed a connection between these two diseases [49,50,51,52,53]. This emphasizes the necessity for well-defined prospective studies with long participant follow-up periods, large sample sizes, study design, and methodology with multi-center and multi-disciplinary collaborations. The groups of included patients must be clearly defined based on specific and well-established diagnostic criteria for both diseases. Ethnic and demographic variations should also be considered, as they may impact the generalizability of the results. Previous studies exploring the association between AD and AMD have yielded controversial findings [26,34,38,39,40,41,42,43,44,45,46,47,48]. Therefore, exploring causal relationships and shared pathogenetic mechanisms in more detail is essential for future research. In a time of technological progress and the introduction of new therapies, the search for reliable biomarkers of AD is becoming increasingly important. Ocular disorders are closely linked to the onset of AD, and recent research is focusing on AD detection using ocular biomarkers and examinations, which are non-invasive, cost-effective, and practical. Diagnosing AD before symptoms manifest is a significant challenge in clinical practice. Currently, diagnosis relies on clinical signs and symptoms, neuroimaging of the brain, and CSF. Additionally, cerebral changes typically occur in the later stages of the disease, after symptoms of brain dysfunction and cognitive decline have already become apparent. Early detection of AD is crucial for timely intervention to slow disease progression and preserve quality of life. Studies have observed the presence of Aβ in the retina of both AD animal models and humans. Moreover, research indicates that Aβ plaques may appear in the retina before they are detectable in the brain, suggesting the potential for effective screening and early detection of AD and dementia through ocular examinations [1,140].

Understanding the mechanisms underlying age-related disease development as the population ages becomes increasingly important and could pave the way for identifying novel therapeutic strategies. At present, immunotherapies have become one of the most promising methods to reverse or slow the progression of AD. Passive immunotherapy targeting key factors in AD pathogenesis offers a promising strategy for effective treatment. Aβ, tau, and neuroinflammation are the primary targets of ongoing immunotherapy investigations. Passive immunotherapy targeting Aβ was initiated decades ago and has achieved significant progress. Currently, two treatments have been approved by the US Food and Drug Administration (FDA): Aducanumab, approved in June 2021, and Lecanemab, approved in July 2023. Both medications are monoclonal antibodies that target aggregated forms of Aβ plaques, removing these deposits from the brains of patients in the early stages of AD. The development of monoclonal antibody drugs targeting tau or immune modulators is still in its early stages with several preclinical and clinical studies showing promising results. For the anti-Aβ strategy, it is important to note that monoclonal antibodies have shown efficacy primarily in patients with early AD. This may be because other factors, such as tau contribute significantly to neuronal loss in the later stages of AD. Therefore, elucidating the clinical effects of anti-Aβ monoclonal antibodies on neuronal loss is crucial. In the future, it would also be worth exploring combined immunotherapy strategies targeting both Aβ and tau in patients with moderate symptoms or medium to severe pathology [141,142]. Regarding AMD treatment, various intravitreal anti-VEGF treatments, including monoclonal antibodies, antibody fragments, and bispecific antibodies, are currently being used in clinical practice for the early stages of the wet and/or neovascular form. The approved anti-VEGF drugs include ranibizumab (Fab fragment), aflibercept (Fc-fusion protein), brolucizumab (scFV fragment), and faricimab (bispecific antibody consisting of an Fab fragment and modified Fc region). Bevacizumab, an anti-VEGF full antibody, is also used off-label to treat wet AMD. These anti-VEGF treatments prevent the promotion of new blood vessel growth, which is a key factor contributing to AMD progression, thereby directly reducing angiogenesis and vascular leakage in the retina. Although antibody-based medications targeting VEGF have revolutionized the treatment of exudative AMD, anti-VEGF therapy is still far from perfect due to pharmacokinetic and compliance issues as well as potential side effects. At this time, there is no effective treatment available to prevent or treat non-exudative AMD. Several therapeutic approaches are being investigated, focusing on disease prevention with antioxidants and visual cycle modulators. This therapy aims to hinder disease progression using drugs with anti-inflammatory impact, influencing oxidative stress levels, acting as a mitochondrial enhancer, β-amyloid inhibitor, and having neuroprotective properties. Efforts to restore vision through cell and gene therapy are also underway [143]. Additionally, treatment modalities developed for AD may find application in AMD. Immunotherapy targeting Aβ has proven effective in safeguarding the RPE epithelium. Likewise, apolipoprotein E, known for its potential in facilitating Aβ degradation and potentially halting AD progression, also shows promise in disrupting drusen formation in AMD. Furthermore, antioxidant-rich dietary supplements have demonstrated efficacy in decelerating the progression of both diseases. Additionally, other mechanisms mentioned may serve as targets for future treatment modalities [17].

Presented findings suggest that identifying genetic predisposing factors for AD and AMD may also contribute to developing new strategies for early diagnosis, monitoring, and treatment of these conditions in an increasingly aging population [13]. Further biological investigations are required to elucidate the underlying mechanism of this association [34]. The genetic similarities discussed present opportunities for diagnostic approaches and customizing therapeutic agents for both AD and AMD. Potential treatment approaches for both diseases include neuroprotection, inhibition of plaque formation, modulation of inflammation, and oxidative stress. A multidisciplinary approach aims to advance the clinical understanding and treatment of retinal diseases, which, in turn, has reciprocal benefits for understanding and addressing brain pathology [13].

## 7. Conclusions 

Despite extensive studies investigating their connections, a definitive association between AMD and the incidence of dementia or AD has not yet been established. Presently, clinical correlations between them remain inconsistent. The variability in current observational findings may be attributed to differences in study populations, designs, AMD subtypes, and other factors across various studies.

To establish the genetic link between AD and AMD, it is essential to conduct comprehensive multi-sample studies encompassing complete clinical datasets from diverse populations. These studies should also investigate the interplay between genetic and environmental factors. Certain key risk factors associated with the development of both diseases are known to be influenced, at least partially, by genetic mechanisms. Moreover, while evidence suggests a potential causality between AD and AMD via shared genetic factors, understanding the source of this pleiotropy and the direction of this association is important. Additionally, further biological experimentation is warranted to elucidate the underlying mechanisms of this intriguing connection. Identifying common genetic and epigenetic characteristics and shared pathogenetic pathways in both conditions will greatly enhance research aimed at prevention, early diagnosis, successful treatment, and ultimately improving the patient’s quality of life.

## Figures and Tables

**Table 1:** Similarities and differences between Alzheimer’s disease and age-related macular degeneration.

Characteristics	Alzheimer’s Disease	Age-Related Macular Degeneration
Risk factors	aging, genetic susceptibility, female sex, atherosclerosis, hypertension, diabetes, obesity, unhealthy diet, smoking, sleep disturbances, traumatic brain injury, social isolation	aging, genetic susceptibility, female sex, atherosclerosis, hypertension, diabetes, obesity, unhealthy diet, smoking, UV light, obstructive sleep apnea, sleep disturbances
Histopathological findings	senile plaques: extracellular Aβ deposits neurofibrillary tangles: intracellular accumulation of p-Tau protein and vitronectin	drusen: focal extracellular deposits, accumulate between the RPE and Bruch’s membrane containing Aβ, clusterin, vitronectin, amyloid P, apolipoprotein E, complement regulatory proteins, and inflammatory mediators
Aβ protein	the main component of senile plaques	component of subretinal drusen
p-Tau deposits	component of neurofibrillary tangles	mildly increased in the RPE
Oxidative and metabolic stress	associated with mental deficiencies and neuronal damage	associated with visual cycle and drusen formation
Glial reactivity	astrocytes and microglia around senile plaques	microglia in the subretinal space and surrounding the drusen
Non-visual disturbances	cognitive deficits (memory loss, aphasia, apraxia, agnosia), depression, behavioral disturbances, inability for self-care	decreased verbal fluency and verbal memory, reduced visuospatial processing and attention, cognitive decline, higher risk of dementia
Cause of visual disturbances	the loss of GCC	photoreceptor layer damage
Location of retinal damage	GCC, RNFL	RPE, photoreceptors
Location of brain damage	atrophy of brain frontal, temporal, and parietal lobes, entorhinal cortex, amygdala, and hippocampus	optic tract volume reduction loss of cerebral white matter connectivity in areas responsible for verbal fluency and memory
Diagnostic procedures	clinical examination, neuropsychological testing CSF sampling brain imaging: MRI, fMRI, CT PET-CR, amyloid imaging	ophthalmological examination OCT, OCTA FA, ICGA
OCT findings	reduced GCC, GC-IPL, peripapillary RNFL	nonexudative AMD: drusen under the RPE, PED exudative AMD: subretinal fluid, CNV
Pleiotropic genes	APOC1–increased risk APOE2 allele–reduced risk APOE4 allele–increased risk	APOC1–increased risk APOE2 allele–increased risk APOE4 allele–reduced risk

UV: ultraviolet; Aβ: amyloid β; p-Tau: hyperphosphorylated tau; RPE: retinal pigment epithelium; GCC: ganglion cell complex; RNFL: retinal nerve fiber layer; MRI: magnetic resonance imaging; fMRI: functional MRI; CT: computed tomography; PET: positron emission tomography; CSF: cerebrospinal fluid; OCT: optical coherent tomography; OCTA: OCT angiography; FA: fluorescein angiography; ICGA: indocyanine green angiography; GC-IPL: macular ganglion cell-inner plexiform layer; PED: pigment epithelial detachment; CNV: choroidal neovascularization; APOC1: Apolipoprotein C-1 gene; APOE: Apolipoprotein E gene.

**Table 2:** Studies investigating the relationship between Alzheimer’s disease and age-related macular degeneration.

Positive Correlations				
Author (Year) Ref.	Study Design	Country	Sample Size	Main Findings
Klaver et al. (1999) [38]	Prospective population-based study	Netherlands	1438 participants; 627 with AMD 811 without AMD	Increased risk of AD among patients with advanced AMD (RR: 2.1). The risk decreased after adjustment for smoking and atherosclerosis (RR = 1.5)
Woo et al. (2012) [39]	Case–control study	South Korea	170 AMD 190 non-AMD patients	Patients with AMD, especially those with geographic atrophy had a greater risk of cognitive impairment compared with non-AMD controls. The severity of AMD was positively correlated with the worsening of cognition function. AMD patients with poor VA have a six times greater risk of mild cognitive impairment compared with AMD patients with good or moderate VA.
Logue et al. (2013) [40]	Genome-wide association study	Multicentric	ADGC sample: 11,840 cases and 10,931 controls from 15 different studies AMDG sample: >7600 cases and 50,000 controls from 14 different studies	Genetic overlap between late-onset AD and AMD. Genetic variants located on chromosome 7 (PILRA, ZCWPW1, ABCA7) linked to AD also contribute to AMD. Shared biological pathways and mechanisms in the development of AD and AMD indicate potential common molecular underpinnings (HGS, involved in the clathrin-mediated endocytosis signaling pathway; TNF, involved in atherosclerosis signaling pathways and LXR/RXR activation pathways).
Tsai et al. (2015) [41]	Longitudinal case–control study	Taiwan	4993 patients newly diagnosed with AMD and 24,965 controls	AMD, particularly nonexudative AMD, was independently associated with an increased risk of AD or senile dementia (HR: 1.55).
Lee et al. (2019) [42]	Retrospective cohort study	USA	3877 participants with 792 AD cases	Ophthalmic conditions such as glaucoma, diabetic retinopathy, and AMD were associated with an increased risk of AD (HR: 1.50).
Rong et al. (2019) [26]	Meta-analysis	Multicentric	21 studies with 7,876,499 study subjects	Increased risk of AMD among patients with cognitive impairment and AD. Increased risk of cognitive impairment and AD among patients with AMD. Patients with AMD had poorer cognitive function compared to controls.
Choi et al. (2020) [43]	Retrospective cohort study	South Korea	308,340 participants	Patients with AMD had a higher risk of AD, with a 1.48-fold higher incidence, even among those with healthy lifestyle behaviors.
Tan et al. (2020) [34]	Genome-wide association study	Multicentric	Data from the Gene Expression Omnibus	Using FUMA, co-localization analysis, and weighted gene co-expression network analysis, 10 genes on chromosome 7, 10 pathways, and 105 biological processes were found to be associated with AD and AMD. This suggests that these 10 genes and the hub genes of these modules associated with shared pathophysiological pathways could potentially serve as diagnostic markers for both diseases.
Hwang et al. (2021) [44]	Population-based prospective study	USA	3375 participants	Increased risk of all types of dementia and AD is associated with AMD. AMD is linked to an 87% greater risk of AD.
Wen et al. (2021) [48]	Population-based retrospective cohort study	Taiwan	10,578 newly diagnosed patients with AMD and 10,578 non-AMD individuals	AMD patients have a 1.23 times higher risk of developing AD. Early onset of AMD correlates with an increased probability of AD development.
Le et al. (2022) [45]	Prospective multicenter randomized study	USA	3157 participants	Cognitive impairment is associated with an increased risk of AMD at 5 years (HR = 1.24) and 10 years (HR = 1.20).
Shang et al. (2023) [46]	Population-based cohort study	UK	2304 cases of dementia	AMD is linked to a heightened risk of dementia. Individuals with AMD and any systemic disease have a higher incidence of dementia compared to either AMD or a systemic disease alone. AMD and diabetes present the highest risk for incident dementia.
Zhang et al. (2024) [47]	Genome-wide association study	China	17,008 patients with AD and 30,178 patients with AMD	The study confirmed genetic pleiotropy between AD and AMD, identifying APOC1 and APOE as pleiotropic genes. ZNF131, ADNP2, and HINFP were identified as novel diagnostic biomarkers for AD and AMD.
Negative Correlations				
Author (Year) Ref.	Study Design	Country	Sample Size	Main Findings
Keenan et al. (2014) [49]	Retrospective cohort study	UK	65,894 participants with AMD, 168,092 participants with dementia	People admitted to the hospital for AMD do not have an increased risk of subsequently developing dementia or AD. No evidence has been found linking AMD to an increased risk of developing dementia or AD.
Schwaber et al. (2020) [50]	Cross-sectional study	USA	157 autopsy ocular and brain specimens	Histopathologic findings failed to support an increased prevalence of AD among patients with AMD.
Kuźma et al. (2021) [51]	Meta-analysis	Multicentric	2559 participants	No evidence of a significant association between AD and AMD based on pooled estimation.
Jiang et al. (2022) [52]	Two-sample bidirectional Mendelian randomization study	Multicentric	NA	The genetic predisposition for advanced AMD did not show a statistically significant causal association with the risk of AD. Reverse Mendelian randomization analysis provided limited evidence supporting a causal effect of AD on advanced AMD.

AD: Alzheimer’s disease; AMD: age-related macular disease, VA: visual acuity; RR: relative risk; ADGC: Alzheimer Disease Genetics Consortium; AMDG: Age-related Macular Degeneration Genetics Consortium; UK: The United Kingdom, HR: hazard ratio; USA: The United States of America; NA: not applicable.

**Table 3:** Main genes, their physiological role, and the possible molecular mechanisms involved in the neuropathology of AMD.

Pathway	Gene	Polymorphism	References
Immune response and complement genes	CFH	rs1061170, rs10922109, rs121913059	[57,61]
	C3	rs2230199, rs1047286	[65]
	CFB	rs641153, rs415667	[67]
	C9	rs62358361, rs34882957	[57,68]
other	PLEKHA/ARMS2/HTRA-1	rs10490924, rs11200638	[74,77]
Oxidative stress genes	MnSOD	Ala-9Val, Ile58Thr	[78,80]
	HMOX1 HMOX2	rs2071747, rs2270363	[82]
Lipid metabolism genes	ApoE	rs429358	[57]
	ABCA1	rs2740488	[57]
	LIPC	rs0468017, rs493258, rs2043085	[57]
Neovascularisation genes	VEGF	rs943080	[76]

**Table 4:** Main genes, their physiological role, and the possible molecular mechanisms involved in the neuropathology of AD.

Gene	Physiological Role	Molecular Mechanisms Implicated in AD	Ref.
PSEN1	Presenilin 1 plays an essential role in neural progenitor maintenance, neurogenesis, neurite outgrowth, synaptic function, neuronal function, myelination, and plasticity.	Presenilin mutations are the main cause of familial Alzheimer’s disease. From a genetic point of view, these mutations seem to result in a gain of toxic function; however, biochemically, they result in a partial loss of function in the γ-secretase complex, which affects several downstream signaling pathways.	[89]
PSEN2	Presenilin 2 processes proteins that transmit chemical signals from the cell membrane into the nucleus. Once in the nucleus, these signals activate genes that are important for cell growth and maturation.	AD-related presenilin mutations can alter intracellular calcium signaling, which leads to Aβ aggregation to form brain plaques and neuronal cell death.	[89]
APOE	The major component of HDL-like particles in the brain, ApoE facilitates the transfer of cholesterol and phospholipids between cells. ApoE serves as a ligand in the receptor-mediated endocytosis of HDL-like particles through the LDL receptor family.	ApoE–lipoproteins bind to several cell-surface receptors to deliver lipids and also to hydrophobic amyloid-β (Aβ) peptide, which is thought to initiate toxic events that lead to synaptic dysfunction and neurodegeneration in AD.	[90]
CLU	Role of CLU in regulating several essential physiological processes, including programmed cell death, metastasis, invasion, proliferation, and cell growth	CLU or apolipoprotein J transporter, can be linked to AD, causing oxidative stress. Therefore, its activity can affect various functions involving complement system inactivation, lipid transport, chaperone activity, neuronal transmission, and cellular survival pathways.	[92]
ADAM10	It is the most important α-secretase in the brain and contributes to the non-amyloidogenic pathway of APP metabolism.	Alteration in APP metabolism (through non-amyloidogenic pathway), synaptic plasticity, and hippocampal neurogenesis.	[91]
BIN1	Participates in immune response, calcium homeostasis, apoptosis, endocytosis of synaptic vesicles, and plasma membrane dynamic.	Contributes to Amyloid (through β-secretase activity) and Tau pathology and is associated with inflammation, apoptosis, and calcium homeostasis.	[95]
APP	APP has important physiological functions during brain development and in neuronal plasticity, memory, and neuroprotection in the mature and aging brain.	APP encodes amyloid precursor protein, a transmembrane protein that is cleaved to form amyloidogenic Aβ peptides. Mutations in APP are associated with familial forms of early-onset Alzheimer’s disease as well as with Cerebral Amyloid Angiopathy (CAA).	[92]
CD2AP	Regulates actin cytoskeleton and membrane trafficking through endocytosis and cytokinesis.	Associated with increased Aβ production, Tau neurotoxicity, abnormal modulation of the neurite structure, and altered integrity of the BBB.	[97]
PICALM	Modulates autophagy, membrane metabolism, internalization of cell receptors, synaptic transmission, removal of apoptotic cells, and endocytic pathways for APP processing.	Associated with increased Aβ production, Tau neurotoxicity, abnormal modulation of the neurite structure, and altered integrity of the BBB.	[92]
ZCWPW1	Regulation of the DNA metabolic process. Additionally, it is involved in epigenetic modulation.	Suppresses insulin resistance. It may activate the PI3K signaling pathway in neurons.	[103]
SPI1	Regulates the immune response and learning-related neuronal activity in the cerebral cortex	Alters the microglial phenotype and transcriptome involving interferon pathways.	[93]
CD33	Involved in the inhibition of immune cell function and cytokine production.	Modulates microglial activation (neuroinflammation) and Aβ clearance through microglial cells.	[94]
TREM2	This microglia receptor regulates proliferation.	Modulates microglial activation (neuroinflammation) and Aβ clearance through microglial cells.	[96]
SLC10A2	Has an important role in encoding the sodium/bile acid cotransporter, as well as in cholesterol metabolism.	Associated with LOAD by dysfunctional cholesterol metabolism, neuronal death, memory impairment, and increased Aβ generation.	[102]

**Table 5:** Epigenetic impacts in AD and AMD.

Target	Disorder	Effect and Epigenetic Impact	Tissue	Refs.
GSTM1, GSTM5 gene	AMD	Reduced expression; Hypermethylation in GSTM1 promoter	retinal pigment epithelial (RPE) cells	[106]
IL17RC gene	AMD	Increased expression; hypomethylation	peripheral blood, retina, and choroid	[107]
HIF1α, VEGF genes	AMD	Reduced expression; Histone deacetylase inhibitor (HDACi	Retinal endothelial cells	[111]
SIRT1 gene	AMD	Reduced expression; Histone deacetylase inhibitor (HDACi)	RPE cells	[114]
CRTC1 gene	AD	Increased expression; Hypomethylation	human hippocampus	[119]
Global DNA	AD	Age-related hypomethylation	Mouse J20 model dentate gyrus and cornu ammonis	[24]
Global DNA	AD	hypermethylation	3xTg–Alzheimer’s disease model	[122]
Transcriptional activation of memory and learning-related genes	AD	overexpression of histone deacetylase 2	Mouse dendritic spine	[124,127]
Histone H2AX	AD	phosphorylated at Ser139	Human hippocampal astrocytes	[126]
Histone H3K27	AD	lysine H3K27 acetylation	entorhinal cortex	[128]
Histones	AD	Decreased acetylation	temporal lobes of the brains of Alzheimer’s subjects	[129]
Histones	AD	Increased acetylation	postmortem brains of Alzheimer’s disease patients	[130]
Histone H3	AD	increased acetylation and phosphorylation	Tg2576 mice; prefrontal cortex	[131]
Hhistone H4	AD	Increased acetylation	Tg2576 mice CA1 region of the hippocampus	[131]
miRNA-9, miRNA-125b, miRNA-146a, and miRNA-155	AMD	dysregulation	retina	[134]
	AD	dysregulation	Age-related degeneration of the brain	
miRNA-7 and the Let-7 cluster, miRNA-23a and the miRNA-27a cluster, miRNA-9, miRNA-34a, miRNA-125b, miRNA-155, and miRNA-146a	AMD	dysregulation	pooled macular tissue	[135]
	AD	dysregulation	pooled temporal lobe neocortex	
miR-9, miR-23a, miR-27a, miR-34a, miR-146a, and miR-155	AMD	dysregulation	Serum of AD patient	[37]
	AD	dysregulation	Serum of AMD patients	
